# High flow nasal cannula oxygen vs. bag-valve-mask for preoxygenation before intubation in patients with hypoxemic respiratory failure - a prospective randomized trial

**DOI:** 10.1186/2197-425X-3-S1-A934

**Published:** 2015-10-01

**Authors:** M Simon, D Frings, C Wachs, S Braune, S Kluge

**Affiliations:** University Medical Center Hamburg-Eppendorf, Department of Intensive Care Medicine, Hamburg, Germany

## Introduction

Scheduled endotracheal intubation for elective surgery is generally considered a safe procedure. In contrast, critically ill patients in the intensive care unit (ICU), especially those with respiratory failure, have an increased risk for hypoxemia-related complications. An increasingly used means of oxygen delivery is its application via high flow nasal cannula (HFNC).

## Objectives

This study was conducted to compare HFNC with bag-valve-mask (BVM) for preoxygenation prior to intubation in patients with hypoxemic respiratory failure.

## Methods

Prospective randomized trial randomizing 40 critically ill patients with hypoxemic respiratory failure to receive either HFNC or BVM for preoxygenation before intubation in the ICU.

## Results

Mean PaO_2_/FiO_2_ at baseline was 200 ± 57 mm Hg in the HFNC group and 205 ± 59 mm Hg in the BVM group (p = 0.76). Mean SpO_2_ at baseline was 96 ± 3 % in the HFNC group and 94 ± 4 % in the BVM group (p = 0.24). The mean lowest oxygen saturation measured by pulse oximetry (SpO_2_) during intubation was 89 ± 18 % in the HFNC group and 86 ± 11 % in the BVM group (p = 0.56). During the one minute of apnea after the induction of anesthesia, SpO_2_ dropped significantly in the BVM group (p = 0.001), while there was no significant difference in the HFNC group (p = 0.17). There were no significant differences between the two groups at any of the pre-defined time points concerning SpO_2_, PaO_2_/FiO_2_, PaCO_2_, heart rate and mean arterial pressure.

## Conclusions

Preoxygenation using HFNC prior to intubation was safe in critically ill patients with hypoxemic respiratory failure. There were no significant differences in gas exchange before and after intubation comparing the HFNC group and the BVM group. However, in contrast to the BVM group there was no significant decrease in SpO_2_ during the apnea phase prior to intubation within the HFNC group.

